# Blood flow dynamics with four-dimensional flow cardiovascular magnetic resonance in patients with aortic stenosis before and after transcatheter aortic valve replacement

**DOI:** 10.1186/s12968-021-00771-y

**Published:** 2021-06-28

**Authors:** Hirokazu Komoriyama, Kiwamu Kamiya, Toshiyuki Nagai, Noriko Oyama-Manabe, Satonori Tsuneta, Yuta Kobayashi, Yoshiya Kato, Miwa Sarashina, Kazunori Omote, Takao Konishi, Takuma Sato, Shingo Tsujinaga, Hiroyuki Iwano, Yasushige Shingu, Satoru Wakasa, Toshihisa Anzai

**Affiliations:** 1 Department of Cardiovascular Medicine, Faculty of Medicine and Graduate School of Medicine, Hokkaido University, Kita 15, Nishi 7, Kita-ku, Sapporo, Hokkaido 060-8638 Japan; 2 Department of Diagnostic and Interventional Radiology, Hokkaido University Hospital, Kita 14, Nishi 5, Kita-ku, Sapporo, Hokkaido 060-8648 Japan; 3 Department of Cardiovascular and Thoracic Surgery, Faculty of Medicine and Graduate School of Medicine, Hokkaido University, Kita 15, Nishi 7, Kita-ku, Sapporo, Hokkaido 060-8638 Japan

**Keywords:** Four-dimensional flow cardiovascular magnetic resonance, Aortic stenosis, Transcatheter aortic valve replacement, Blood flow dynamics

## Abstract

**Background:**

Pre- and post-procedural hemodynamic changes which could affect adverse outcomes in aortic stenosis (AS) patients who undergo transcatheter aortic valve replacement (TAVR) have not been well investigated. Four-dimensional (4D) flow cardiovascular magnetic resonance (CMR) enables accurate analysis of blood flow dynamics such as flow velocity, flow pattern, wall shear stress (WSS), and energy loss (EL). We sought to examine the changes in blood flow dynamics of patients with severe AS who underwent TAVR.

**Methods:**

We examined 32 consecutive severe AS patients who underwent TAVR between May 2018 and June 2019 (17 men, 82 ± 5 years, median left ventricular ejection fraction 61%, 6 self-expanding valve), after excluding those without CMR because of a contraindication or inadequate imaging from the analyses. We analyzed blood flow patterns, WSS and EL in the ascending aorta (AAo), and those changes before and after TAVR using 4D flow CMR.

**Results:**

After TAVR, semi-quantified helical flow in the AAo was significantly decreased (1.4 ± 0.6 vs. 1.9 ± 0.8, *P* = 0.002), whereas vortical flow and eccentricity showed no significant changes. WSS along the ascending aortic circumference was significantly decreased in the left (*P* = 0.038) and left anterior (*P* = 0.033) wall at the basal level, right posterior (*P* = 0.011) and left (*P* = 0.010) wall at the middle level, and right (*P* = 0.012), left posterior (*P* = 0.019) and left anterior (*P* = 0.028) wall at the upper level. EL in the AAo was significantly decreased (15.6 [10.8–25.1 vs. 25.8 [18.6–36.2]] mW, *P* = 0.012). Furthermore, a significant negative correlation was observed between EL and effective orifice area index after TAVR (r = − 0.38, *P* = 0.034).

**Conclusions:**

In severe AS patients undergoing TAVR, 4D flow CMR demonstrates that TAVR improves blood flow dynamics, especially when a larger effective orifice area index is obtained.

**Supplementary Information:**

The online version contains supplementary material available at 10.1186/s12968-021-00771-y.

## Background

Transcatheter aortic valve replacement (TAVR) has emerged as a minimally invasive treatment option for patients with severe symptomatic aortic stenosis (AS) who cannot tolerate surgical aortic valve replacement (SAVR) and those at intermediate–high surgical risk, and is expanding to younger, lower-surgical-risk patients [1–3].

In patients with AS, chronic pressure overload evolves to left ventricular (LV) remodeling, characterized by cardiac muscle hypertrophy and interstitial collagen deposition, ultimately leading to systolic and/or diastolic dysfunction [4, 5]. While it is known that patients who undergo TAVR can improve diastolic dysfunction and undergo LV reverse remodeling, residual diastolic dysfunction and lack of LV reverse remodeling after TAVR may adversely affect the prognosis [6]. The ascending aorta (AAo), as an immediate buffer of LV ejection, plays important roles in determining LV afterload [7]. In fact, increased arterial stiffness and hemodynamic abnormalities in the AAo increase LV oxygen demand and cardiac work, leading to exacerbation of heart failure (HF) [8, 9]. Thus, non-invasive pre- and post-procedural hemodynamic assessment in the AAo may help evaluate the effects of TAVR.

Time-resolved three-dimensional (3D) phase-contrast cardiovascular magnetic resonance (CMR), also known as four-dimensional (4D) flow CMR, is a novel blood flow imaging technique that allows accurate visualization and quantification of aortic blood flow dynamics [10]. Notably, 4D flow CMR can also calculate wall shear stress (WSS), which is the force acting in a tangential direction to the blood vessel lumen surface due to blood flow, and energy loss (EL), which is the kinetic energy lost in blood flow due to frictional force. Several studies have demonstrated various blood flow patterns assessed by this modality in healthy subjects [11, 12], patients with AS [13], and patients post-SAVR or -TAVR [14–16]. Recently, we have reported a successful case of assessment of blood flow and WSS in the AAo before and after TAVR using 4D flow CMR [17]. Blood flow dynamic parameters obtained with 4D flow CMR, such as WSS and EL in the AAo, are expected to be an important indicator of LV afterload, which would be related to impaired LV expansion and progression of LV remodeling [18]. However, it is uncertain how blood flow dynamics are impaired in patients with AS and how TAVR alters blood flow dynamic parameters obtained by 4D flow CMR. Furthermore, it is unclear which parameters related to blood flow dynamics in the AAo could be improved after TAVR. Accordingly, we sought to investigate the changes in blood flow dynamics before and after TAVR using 4D flow CMR to clarify their functional significance.

## Methods

### Study design

This was a single-center, observational, prospective study that included consecutive patients who underwent TAVR with a diagnosis of severe symptomatic AS according to the updated guidelines [19]. The study protocol was approved by the Ethics Committee of Hokkaido University Hospital (018-0223 and 019-0090). The investigation conformed with the principles outlined in the Declaration of Helsinki. All patients gave informed consent to participate in the study.

### Study population

**Fig. 1:**
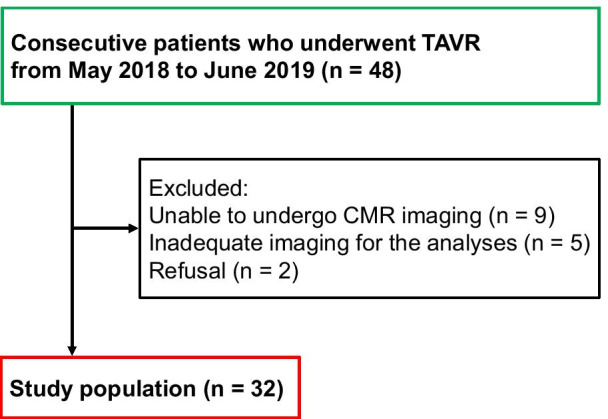
Flow diagram of present study. C*MR* cardiovascular magnetic resonance, *TAVR* transcatheter aortic valve replacement

We originally screened 48 consecutive patients who underwent TAVR between May 2018 and June 2019. All patients enrolled in this study were diagnosed with severe AS. They met any of the following criteria on transthoracic echocardiography: a peak aortic velocity (V_max_) ≥ 4.0 m/s, a mean transaortic pressure gradient (mean PG) ≥ 40 mmHg, an aortic valve area (AVA) ≤ 1.0 cm^2^, or an aortic valve area index (AVAI) ≤ 0.6 cm^2^/m^2^. Among patients with severe AS, those satisfying any of V_max_ ≥ 5.0 m/s, mean PG ≥ 60 mmHg, AVA ≤ 0.6 cm^2^, or AVAI ≤ 0.4 cm^2^/m^2^ criteria were called very severe AS [20]. Of these, patients without CMR because of a contraindication (n = 9) to CMR and those with inadequate imaging unsuitable for analysis because of artifacts (n = 5) were excluded. Two patients declined to participate in this study. Ultimately, 32 patients were included in this study (Fig. 1). In addition, 12 control subjects without significant AS or aortic regurgitation were recruited to compare the blood flow dynamic parameters obtained by 4D flow CMR to the pre- or post-TAVR groups.

### TAVR procedure

All cases were discussed at a meeting of the multidisciplinary Heart Team and were indicated to undergo TAVR as recommended by current guidelines [1]. TAVR was performed through a transfemoral, transapical or transsubclavian route depending on pre-procedural vascular assessment. In most patients, TAVR was performed with transesophageal echocardiographic guidance under general anesthesia. Pre- and post-dilatation was performed at the operator’s discretion.

### Echocardiography measurements

Echocardiography was performed within 2 weeks before and after TAVR, respectively. LV systolic or diastolic function were assessed by echocardiography before and after TAVR: LV end-diastolic dimension (LVEDD), LV end-systolic dimension (LVESD), LV ejection fraction (LVEF), the ratio between early and late diastolic transmitral flow velocity (E/A), the ratio of maximal early diastolic filling wave velocity to maximal early diastolic myocardial velocity (E/e’), and left atrial (LA) volume index (LAVI). LVEF was measured from the apical 4- and 2-chamber images using the biplane method of disks. LA volume was measured from standard apical 4-chamber views at end-systole just before mitral valve opening. The biplane method of disks was used to calculate LA volume. LV mass (LVM) was calculated according to the following formula:

$${\text{LVM}}\, = \,0.{8}\,\, \times \,\left\{ {{1}.0{4}\, \times \,\left[ {\left( {{\text{LVEDD}}\, + \,{\text{LV}}\,{\text{inferolateral}}\,{\text{wall}}\,{\text{thickness}}\, + \,{\text{interventricular}}\,{\text{septum }}\,{\text{thickness}}} \right)^{{3}} \,{-}\,\left( {{\text{LVEDD}}} \right)^{{3}} } \right]} \right\}\, + \,0.{6}\,{\text{g}}$$

LAVI and LVM index (LVMI) were calculated by dividing LA volume or LV mass by body surface area of patients, respectively. Relative wall thickness (RWT) was defined as 2 times inferolateral wall thickness divided by the LVEDD. LV remodeling was assessed based on LVEDD, LVMI and RWT.

After TAVR, the effective orifice area (EOA) was estimated using the LV outflow tract diameter and the LV outflow tract time velocity integral measured immediately proximal to the stent, as recommended previously [21]. The effective orifice area index (EOAI) was calculated with the EOA of the prosthesis divided by the body surface area of patients.

### Cardiovascular magnetic resonance

CMR imaging using a 3 T CMR scanner (Achieva TX, Philips Healthcare, Best, The Netherlands) with a 32-channel phased-array receiver torso-cardiac coil was performed in patients before TAVR (median interval, 24 [1–39] days) and after TAVR (median interval, 6 [4–6] days). Retrospective electrocardiogram gating and a respiratory navigator placed on the lung–liver interface (7 mm window size) were used. In all patients, LV and aortic blood flow dynamics were evaluated using 4D flow CMR measuring 3D blood flow velocities with full volumetric coverage of the heart and the thoracic aorta. 4D flow CMR data were acquired in a sagittal oblique 3D data including the entire heart and thoracic aorta without contrast agent. We acquired 4D flow sequence with 3D spatial encoding. We scanned 4 or 5 mm slice thickness without overlapping using 3D spatial encoding. After data acquisition, we reconstructed images with slice gap 2 or 2.5 mm. The scan parameters were: echo time = 1.73 ms, repetition time = 3.2 ms, flip angle α = 10°, field of view 400 × 400 mm, matrix 256 × 229, in-plane spatial resolution 1.56 × 1.75 mm^2^, slice thickness 4 mm and slice overlapping 2 mm, or slice thickness 5 mm and slice overlapping 2.5 mm (depending on the patient's body size), temporal resolution 12 phases/cardiac cycle, k-space segmentation factor 6, Sensitivity encoding factor R = 3. Velocity encoding timing was TR-interleaved. Partial k-space coverage methods and k–t undersampling were not used. Velocity encoding (VENC) was set to 2.5–8.0 m/s individually based on the peak blood flow velocity in the AAo with a secured margin. The acquisition time of 4D flow CMR was approximately 8–20 min.

Maxwell correction, eddy current correction, and gradient non-linearity correction methods were performed by the CMR scanner. Acquired data including phase-contrast 3-axis cine images, magnitude images and steady-state free procession cine images were processed using commercially available software (iTFlow, Cardio Flow Design Inc., Tokyo, Japan), which visualized the cardiovascular geometry and blood flow [22, 23]. Moreover, WSS and EL were calculated using the software [22].

### Data analysis

**Fig. 2:**
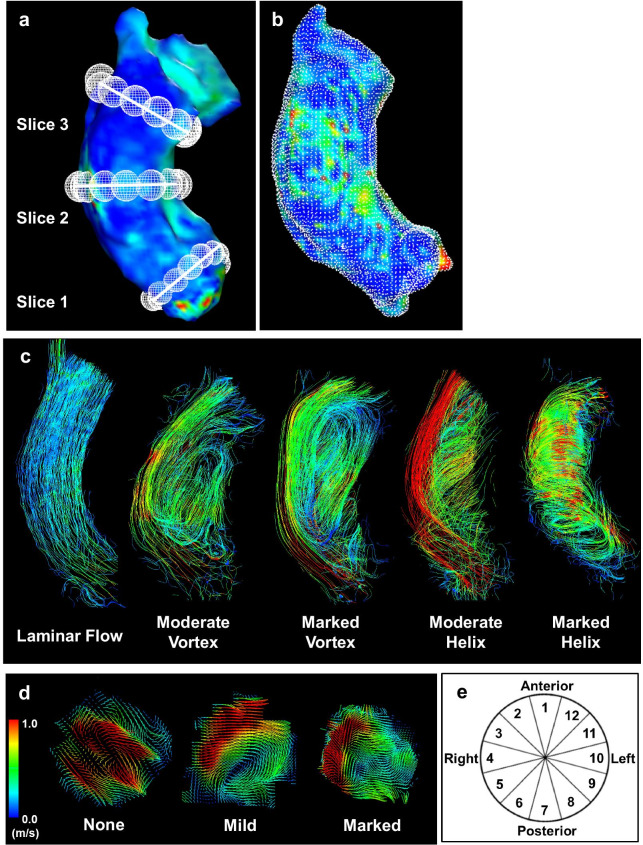
Methodological diagrams. **a** Position of analysis planes in the AAo at the level of the sinotubular junction (Slice 1), mid AAo (Slice 2), and proximal to the brachiocephalic trunk (Slice 3). **b** WSS calculation in the entire AAo. WSS is calculated at each white dot. **c** Laminar flow and examples of grading for vortical and helical flow. **d** Peak systolic blood flow velocity map at the level of the mid AAo exhibits blood flow eccentricity. **e** Cross section of the AAo shows segments along the aortic wall circumference. *AAo* ascending aorta, *WSS* wall shear stress

The blood flow pattern in the AAo was evaluated as previously described [14]. Three analysis planes were positioned perpendicular to the aortic wall at the level of the sinotubular junction (Slice 1), mid AAo (Slice 2), and proximal to the brachiocephalic trunk (Slice 3) (Fig. 2a).

Blood flow pattern in the AAo was evaluated using streamlines, which is the instantaneous velocity field at a specific temporal phase. Two readers (one cardiologist and one radiologist) simultaneously observed the streamlines of each phase from the patient's left front. The systolic blood flow pattern was semi-quantitatively evaluated as three grades for vortical flow and helical flow, respectively. Discordant cases were evaluated by a third reader (a radiologist experienced in cardiovascular imaging). Vortical flow was defined as revolving particles around a point within the vessel with a rotation direction deviating by more than 90° from the physiological flow direction [14]. Helical flow was defined as regional fluid circulation along the longitudinal axis of the vessel, thereby creating a corkscrew-like motion [14]. Vortex and helix strengths were graded in three categories: 1 = none (none or almost none), 2 = moderate (obvious. between 1 and 3), 3 = marked (mainstream). The detailed distinction between moderate and marked strengths was as follows. Vortical flow from the brachiocephalic artery bifurcation to the sinotubular junction was determined as marked, and smaller vortical flow was determined as moderate. Helical flow with laminar flow was classified as moderate, and helical flow without laminar flow was classified as marked. Examples of grading for vortical and helical flow are shown in Fig. 2c.

Peak velocity blood flow eccentricity in the mid AAo (Slice 2) during systole was semi-quantitatively evaluated as three grades: 1 = none (if high velocity systolic flow was centrally focused, occupying the majority of the vessel lumen), 2 = mild (if high velocity systolic flow occupied between one- and two-thirds of the vessel lumen), 3 = marked (if high velocity systolic flow occupied one-third or less of the vessel lumen) (Fig. 2d) [14].

Mean and maximum blood flow velocity and WSS in the AAo were calculated using the above-mentioned software tool (iTFlow, Cardio Flow Design Inc.) as described previously [22]. In brief, the surface of the segmented aorta at the moment of peak systole is represented by connected triangles, and the WSS is defined as the spatial gradient of the 3D velocity vector in each edge point, perpendicular to the vessel wall. This algorithm has been established previously by Potters et al. [24]. The WSS was calculated from the formula below (Formula 1).

1 $${\text{WSS}} = \mu \frac{dv}{{dy}}$$

*μ*: blood viscosity (μ = 0.004 Pa s), *v*: blood flow velocity, *y*: distance from the wall.

First, an anatomical segmentation of the AAo is obtained using iTFlow software. The AAo was defined as the aortic segment between the sinotubular junction and the origin of the brachiocephalic trunk before TAVR. After TAVR, the AAo was defined as the segment between the first circumferential area of the AAo not susceptible to metal-induced artifacts and the origin of the brachiocephalic trunk. Subsequently, we determined the 3D WSS over the complete AAo and displayed it on a color-coded map. Peak WSS (WSS peak) used the highest WSS value in this color-coded map of the entire AAo. As the average WSS (WSS average), the mean value of WSS of the entire AAo in the phase in which the peak was recorded is used (Fig. 2b). Furthermore, to investigate changes in WSS in each region, calculation of WSS were performed for 12 segments along the aortic circumference for each analysis plane (Slice 1 to Slice 3) (Fig. 2a and e) [14]. By specifying the position of a mesh sphere whose size can be changed, it is possible to calculate WSS on any surface in contact with the sphere. The WSS value in each segment was the average of the calculated values at multiple contact points (31.9 ± 11.7) between the aorta reconstructed in 3D and the sphere. The assessment of the WSS is shown in Fig. 2a, b, and e.

EL was calculated from the spatial velocity gradient of blood flow and blood viscosity according to the formula below (Formula 2) [22, 25]. EL across the region of interest (left ventricle or AAo) was calculated for each of the 12 phases/cardiac cycles and averaged for systolic and diastolic phases, respectively. Regions of interest were selected for each slice of the coronal image. The heart chamber from the mitral valve to the aortic valve was defined as the region of the LV, and the aorta from the aortic valve to the brachiocephalic artery was defined as the region of the AAo.

2 $$EL = \int {\left( \mu \right)\sum\limits_{ij} {\frac{1}{2}\left( {\frac{{\partial u_{i} }}{{\partial x_{j} }} + \frac{{\partial u_{j} }}{{\partial x_{i} }}} \right)} }^{2} dV$$

*μ*: blood viscosity (μ = 0.004 Pa s), *x*: horizontal direction of the phase image, *u*: horizontal direction component of the blood velocity vector.

### Statistical analyses

Continuous variables are presented as the mean ± standard deviation when normally distributed, and as the median and interquartile range when not normally distributed. Comparisons between the TAVR group and the control group were performed by the Mann–Whitney U-test for continuous variables and by the chi-squared test for dichotomous variables. The changes in the blood flow dynamic parameters after TAVR were evaluated by the Wilcoxon signed-rank test. Inter-reader agreement for blood flow patterns was assessed using quadratic weighted kappa statistics (along with their standard errors). Linear regression models were constructed to assess the relationship between WSS average or WSS peak in the AAo and AVAI before TAVR (or EOAI after TAVR). The same analysis was performed to confirm the relationship between EL and AVAI before TAVR (or EOAI after TAVR). Another linear regression model was constructed to confirm the relationship between WSS or EL in the AAo and the parameters of the LV function or remodeling, as well as the relationship between vortical flow, helical flow, blood flow eccentricity, WSS or EL and aortic size and pre- and post-TAVR aortic pressure gradient. All tests were two tailed, and a *P* value < 0.05 was considered statistically significant. All analyses were performed using Stata MP64 (version 15, Stata Corp, College Station, Texas, USA).

## Results

### Patients’ characteristics

The baseline characteristics of all 32 subjects (82 ± 5 years, 17 (53%) men) are shown in Table 1. Mean transaortic PG was 53 ± 21 mmHg and AVAI was 0.5 ± 0.1 cm^2^/m^2^. Median LVEF was 61% (interquartile range 54–70%), and mean stroke volume index was 47 ± 12 ml/m^2^. Twenty-eight (88%) patients underwent transfemoral TAVR, and a balloon-expanding valve was used in 26 (81%) patients. Eighteen (56%) were classified as very severe AS. There was no significant difference in hemodynamic changes between severe AS and very severe AS groups (Additional file 5: Table S1).

**Table 1:** Baseline characteristics

Variables	n = 32
Age, years	82 ± 5
Male, n (%)	17 (53)
Body mass index, kg/m^2^	21.3 ± 3.8
STS-PROM score, %	5.1 (4.1–7.1)
Past history, n (%)	
Hypertension	23 (72)
Dyslipidemia	20 (63)
Diabetes mellitus	11 (34)
Atrial fibrillation	4 (13)
Coronary artery disease	12 (38)
Stroke	5 (16)
Laboratory data	
Hemoglobin, g/dL	11.5 ± 1.4
Creatinine, mg/dL	0.83 (0.71–1.14)
NT-proBNP, pg/mL	1322 (568–3145)
Echocardiography	
LVEDD, mm	46.7 (43.0–50.0)
LVEF, %	61 (54–70)
SVI, mL/m^2^	46.5 ± 12.4
IVS, mm	12.0 ± 2.0
LVMI, g/m^2^	117.5 (102–141)
LAVI, mL/m^2^	45.5 (39.7–63.0)
E/A ratio	0.70 (0.56–0.97)
E/e’ ratio	13.1 ± 4.8
AVAI, cm^2^/m^2^	0.45 ± 0.11
Mean transaortic PG, mmHg	53 ± 21
Computed tomography	
Aortic annular area, mm^2^	444 (388–480)
Aortic annular perimeter, mm	75.7 (70.7–80.5)
Bicuspid valve, n (%)	2 (6)
Ascending aortic diameter, mm	33.2 ± 3.7
Access route for TAVR, n (%)	
Femoral	28 (88)
Apical	2 (6)
Subclavian	2 (6)
TAVR model, n (%)	
SAPIEN 3	26 (81)
Evolut R	3 (9)
Evolut PRO	3 (9)
THV size, n (%)	
23 mm	11 (34)
26 mm	13 (41)
29 mm	8 (25)

Continuous variables are presented as mean ± standard deviation if normally distributed, and median (interquartile range) if not normally distributed

Categorical variables are presented as number of patients (%)

*AVAI* aortic valve area index; *IVS* interventricular septum; *LAVI* left atrial volume index; *LVEDD* left ventricular end-diastolic dimension; *LVEF* left ventricular ejection fraction; *LVMI* left ventricular mass index; *NT-proBNP* N-terminal pro-brain natriuretic peptide; *PG* pressure gradient; *STS-PROM* Society of Thoracic Surgeons Predicted Risk of Mortality; *SVI* stroke volume index; *TAVR* transcatheter aortic valve replacement; *TAVR* transcatheter aortic valve replacement

The changes in the echocardiographic parameters before and after TAVR are shown in Table 2. EOAI was significantly increased, and the peak and mean transaortic PG were significantly decreased after TAVR. Conversely, there were no significant changes in the echocardiographic data for assessment of LV systolic or diastolic function and remodeling. None of our patients had severe prosthesis–patient mismatch (EOAI < 0.65 cm^2^/m^2^), or paravalvular leakage of 3 degrees or more, which have an adverse prognostic effect after TAVR.

**Table 2:** Changes on hemodynamic and echocardiographic parameters before and after TAVR (n = 32)

Variables	Pre-TAVR	Post-TAVR	*P* value
Heart rate (bpm)	66 (61–76.5)	70.5 (61–79.5)	0.67
Systolic blood pressure (mmHg)	120 (110–127)	118 (110–129.5)	0.33
Diastolic blood pressure (mmHg)	64.5 (57.5–68.5)	60.5 (48.5–71.5)	0.40
Peak aortic velocity (V_max_) (m/s)	4.6 ± 0.8	2.3 ± 0.4	< 0.001
Mean transaortic PG (m/s)	53 ± 21	12 ± 4	< 0.001
AVAI or EOAI (cm^2^/m^2^)	0.5 ± 0.1	1.1 ± 0.2	< 0.001
Paravalvular leak severity I/II/III/IV, n (%)	–	24 (75)/4 (13)/0/0	–
LVEDD (mm)	46.7 (43.0–50.0)	46.0 (42.5–49.0)	0.99
LVESD (mm)	28.0 (24.5–34.5)	28.5 (25.0–32.0)	0.71
LVEF (%)	61 (54–70)	64 (56–70)	0.010
Transmitral peak E/A ratio	0.70 (0.56–0.97)	0.70 (0.62–0.91)	0.058
E/e′ ratio	13.1 ± 4.8	14.0 ± 4.2	0.25
LAVI (mL/m^2^)	45.5 (39.7–63.0)	44.8 (36.1–58.5)	0.78
LVMI (g/m^2^)	118 (102–141)	115 (98–145)	0.65
RWT	0.44 (0.39–0.49)	0.44 (0.38–0.47)	0.63

Continuous variables are presented as mean ± standard deviation if normally distributed, and median (interquartile range) if not normally distributed

Categorical variables are presented as number of patients (%)

*AVAI* aortic valve area index; *EOAI* effective orifice area index; *LAVI* left atrial volume index; *LVEDD* left ventricular end-diastolic dimension; *LVESD* left ventricular end-systolic dimension; *LVEF* left ventricular ejection fraction; *LVMI* left ventricular mass index; *PG* pressure gradient; *RWT* relative wall thickness; *TAVR* transcatheter aortic valve replacement

The characteristics of the control group are shown in Additional file 5: Table S2; there were no significant differences in age and sex between the TAVR group and the control group. Cardiac function was comparable between the groups, with similar LV dimensions and LVEF. However, the TAVR group had a higher LVMI than the control group.

### Blood flow patterns and flow eccentricity in ascending aorta

**Fig. 3:**
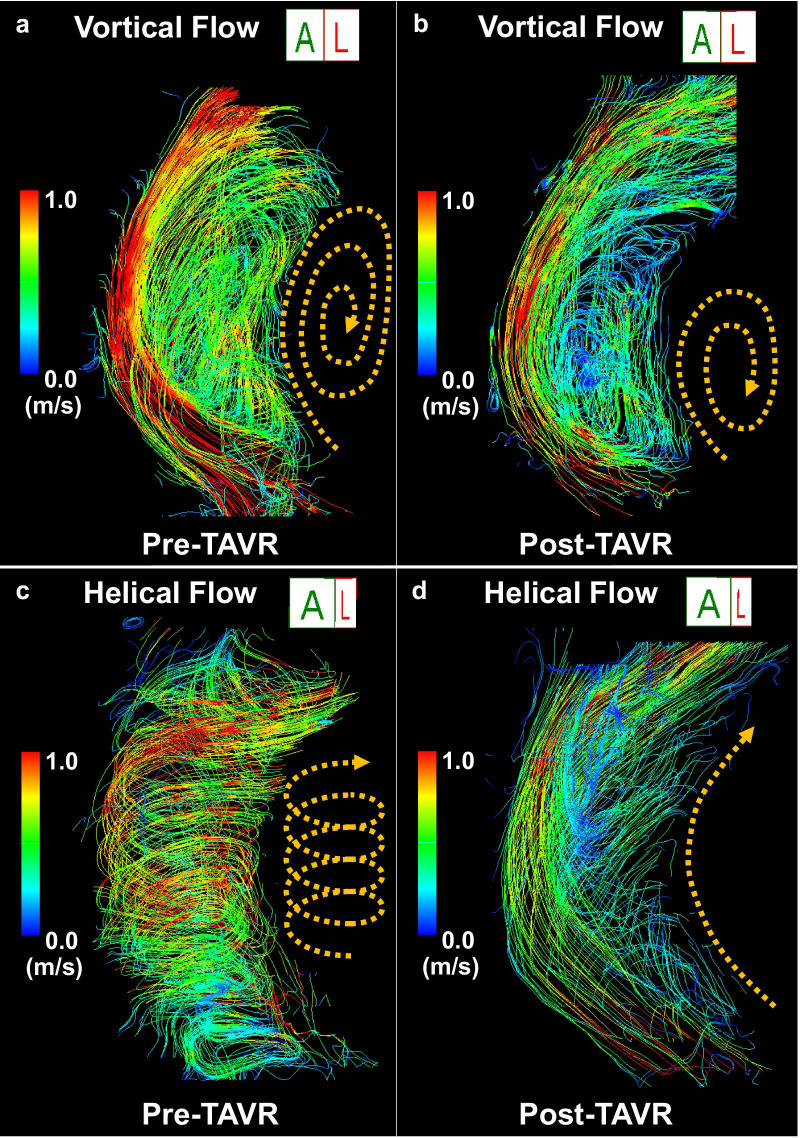
Representative cases. In a patient, **a** marked vortical flow (dotted line and curved arrow) was observed before TAVR, and **b** vortical flow was decreased after TAVR. In another patient, **c** marked helical flow (dotted line and curved arrow) was observed before TAVR, and **d** helical flow disappeared after TAVR. *TAVR* transcatheter aortic valve replacement

Two representative cases which showed significant improvement in vortical or helical blood flow in the AAo following TAVR procedure are demonstrated in Fig. 3 and supplementary videos (see Additional files: 1, 2, 3, and 4 [Videos S1, S2, S3, and S4]).

Of the 64 examinations, the number of discordant cases was 16 for vortex, 11 for helix, and 12 for eccentricity. The quadratic weighted kappa coefficients for inter-observer agreement for blood flow patterns were as follows: k = 0.654; standard error = 0.097 for vortex; k = 0.751, standard error = 0.098 for helix; and k = 0.627, standard error = 0.111 for eccentricity. The reproducibility of each parameter for the blood flow pattern was substantial.

Semi-quantified flow eccentricity values were significantly higher in the pre- and post-TAVR groups compared to the control group. Helical blood flow values were higher in the pre-TAVR group compared to the control group; however, there was no significant difference after TAVR. Furthermore, there was no significant difference in the degree of vortical blood flow between the pre- or post-TAVR groups and the control group (Additional file 5: Table S3).

**Fig. 4:**
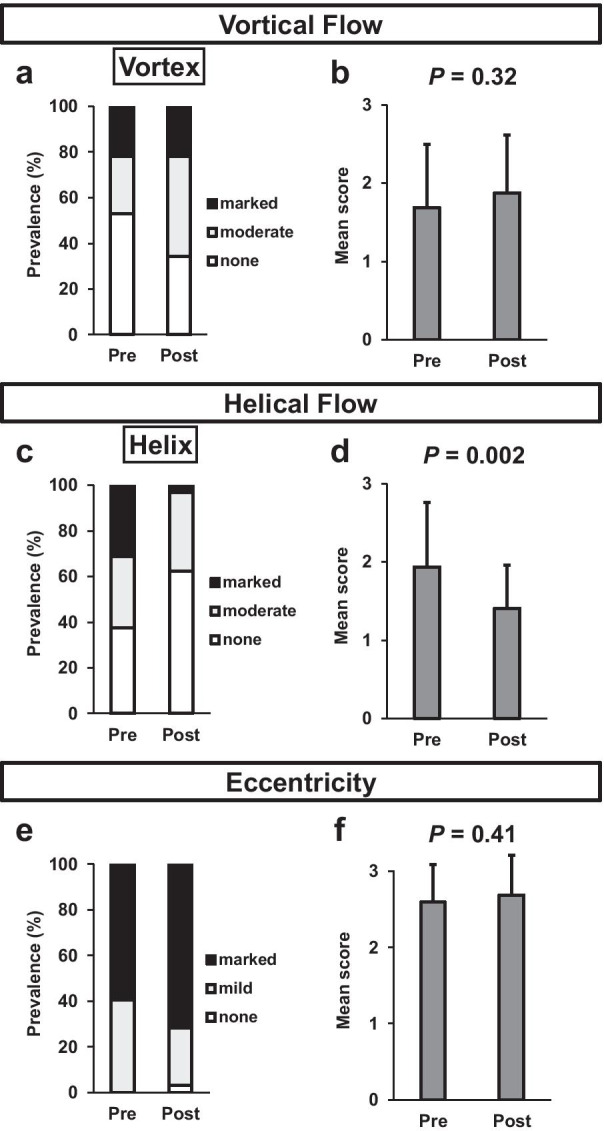
Prevalence of each grade of **a** vortical flow and **b** its mean score, **c** helical flow and **d** its mean score, and **e** blood eccentricity and **f** its mean score, before and after TAVR. *TAVR* transcatheter aortic valve replacement

After TAVR, semi-quantified helical blood flow in the AAo significantly decreased, whereas vortical blood flow and flow eccentricity showed no significant changes (Fig. 4).

### Wall shear stress in ascending aorta

WSS average and WSS peak in the entire AAo were significantly higher in the pre- and post-TAVR groups compared to the control group (Additional file 5: Table S3).

**Fig. 5:**
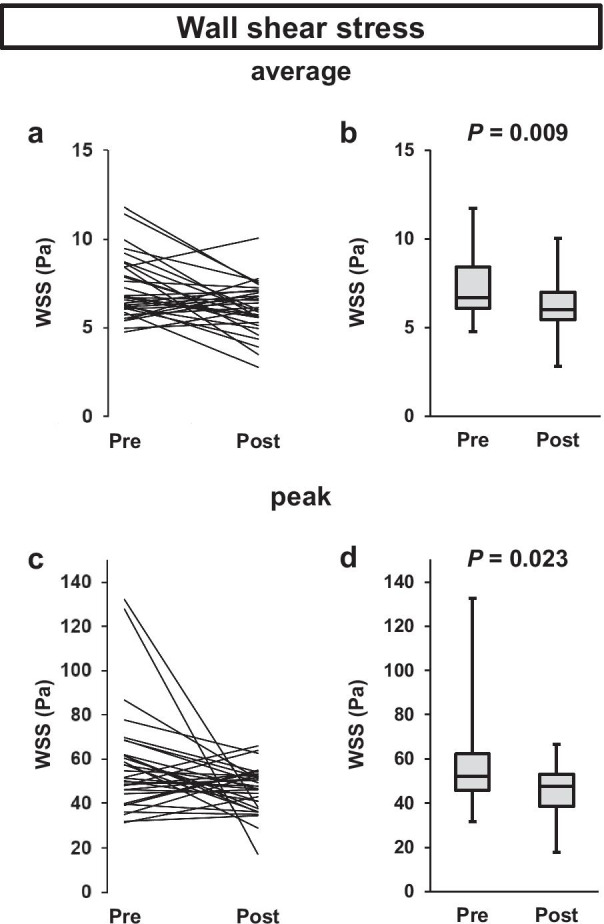
WSS average (**a** and **b**) and WSS peak (**c** and **d**) in the entire AAo before and after TAVR. *AAo* ascending aorta, *TAVR* transcatheter aortic valve replacement, *WSS* wall shear stress

The changes of WSS average and WSS peak in the entire AAo after TAVR are shown in Fig. 5. WSS average and WSS peak significantly decreased from 6.7 [6.1–8.4] Pa to 6.0 [5.4–7.0] Pa (*P* = 0.009) and from 52.0 [45.6–62.2] Pa to 47.5 [38.4–53.1] Pa (*P* = 0.023), respectively. The relationship between WSS at pre- or post-TAVR and AVAI or EOAI is shown in Additional file 5: Table S4. There was no significant correlation between WSS average or WSS peak and AVAI before TAVR. There was also no significant correlation between WSS average or WSS peak and EOAI after TAVR.

**Fig. 6:**
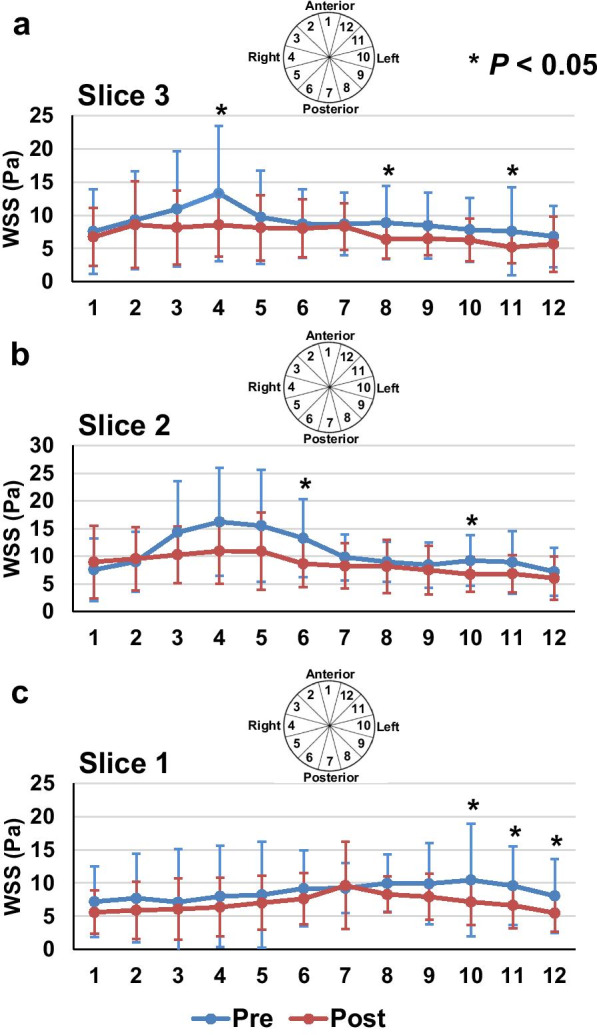
Distribution of peak WSS in 12 segments along aortic circumference for **a** slice 3, **b** slice 2 and **c** slice 1 before and after TAVR. *P < 0.05. *TAVR* transcatheter aortic valve replacement, *WSS* wall shear stress

Figure 6 shows peak WSS in 12 segments along the aortic circumference for analysis in plane slices 1 to 3 before and after TAVR. WSS was significantly decreased in the left and left anterior wall at the basal level, right posterior and left wall at the middle level, and right, left posterior and left anterior wall at the upper level.

### Energy loss in ascending aorta and left ventricle

The systolic EL in the AAo was significantly higher in the pre-TAVR group compared to the control group; however, there was no significant difference after TAVR (Additional file 5: Table S3).

**Fig. 7:**
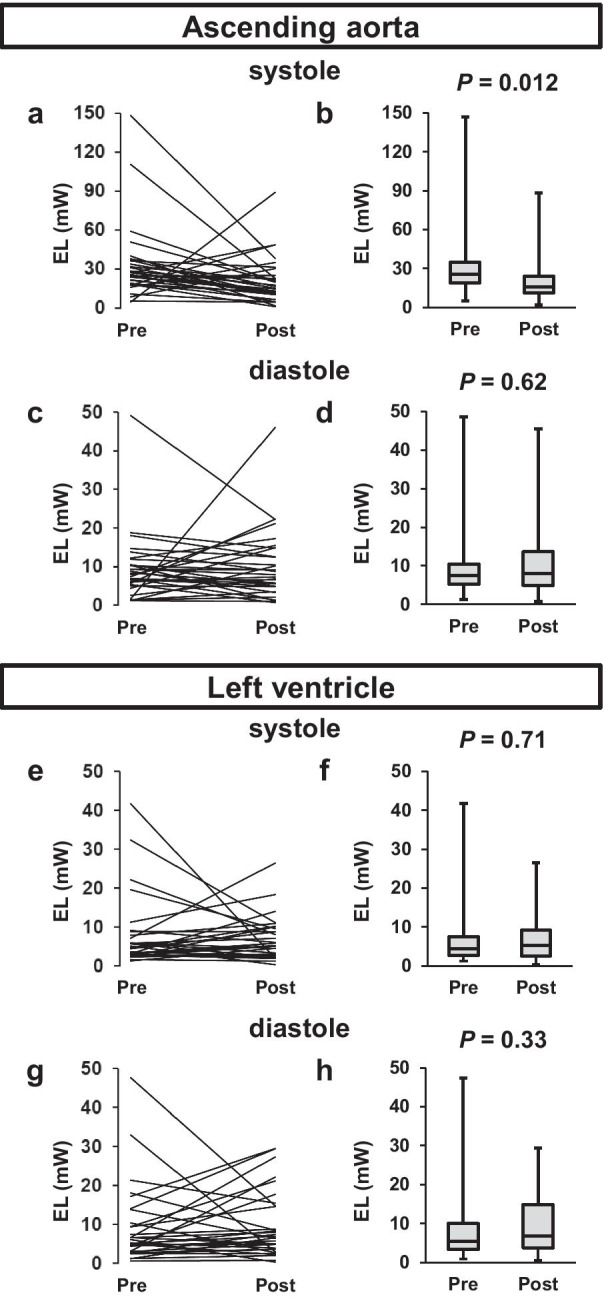
EL in the AAo before and after TAVR during **a** and **b** systole and **c** and **d** diastole. EL in LV before and after TAVR during **e** and **f** systole and **g** and **h** diastole. *AAo* ascending aorta, *EL* energy loss, *LV* left ventricle, *TAVR* transcatheter aortic valve replacement

**Fig. 8:**
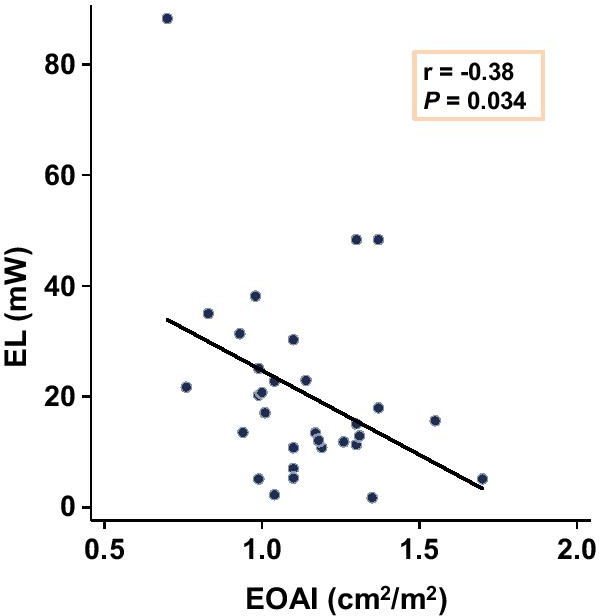
Linear regression model presenting relationship between systolic EL in the AAo and EOAI after TAVR. *AAo* ascending aorta, *EL* energy loss, *EOAI* effective orifice area index, *TAVR* transcatheter aortic valve replacement

The changes of EL in the AAo and LV before and after TAVR is shown in Fig. 7. EL in the AAo during the systolic phase was significantly decreased (15.6 [10.8–25.1 vs. 25.8 [18.6–36.2]] mW, *P* = 0.012), while that in the LV was unchanged after TAVR. Furthermore, a significant negative correlation was observed between EL in the AAo during the systolic phase and EOAI after TAVR (r = − 0.38, *P* = 0.034, Fig. 8). However, there was no significant correlation between EL in the AAo during the systolic phase and AVAI before TAVR (Additional file 5: Table S4). A significant positive correlation was observed between systolic EL in the AAo and mean transaortic pressure gradient after TAVR, and between diastolic EL in the AAo and the AAo diameter after TAVR.

### Differences between the self- and the balloon-expanding bioprostheses

Six subjects received self-expanding TAVR bioprostheses. There were no significant differences on blood flow dynamic parameters in 4D flow CMR after TAVR between self-expanding and balloon-expanding bioprostheses (n = 26) (Additional file 5: Table S5). However, the self-expanding bioprosthesis group had significantly greater decrease in WSS average after TAVR than the balloon-expanding bioprosthesis group. In addition, systolic EL in the AAo tended to be decreased in the self-expanding bioprosthesis group compared with the balloon-expanding bioprosthesis group (Additional file 5: Table S6).

## Discussion

This study is an initial report evaluating the changes of blood flow dynamics using 4D flow CMR before and after TAVR. The major findings of this study were that (1) helical blood flow, WSS and EL in the AAo were significantly decreased, and (2) EL in the AAo was negatively correlated with EOAI after TAVR.

### Flow patterns

Using 3D phase-contrast cine CMR, Kilner et al. observed that helical blood flow develops in the AAo and extends towards the aortic arch in healthy subjects [12]. Segadal and Matre also observed a bidirectional velocity profile in late systole and early diastole, featuring retrograde velocity (i.e., vortical blood flow) along the inner wall and sustained antegrade flow along the outer wall [26]. Previous studies have demonstrated the role of helical blood flow in the AAo, showing that relatively coherent swirling of blood might avoid excessive dissipation of energy by limiting flow instability in arteries [27, 28]. Although helical blood flow plays a positive physiological role in enhancing blood flow transport, the flow pattern changes when aortic morphological changes occur, such as aortic enlargement or AS [13, 29]. Vortical flow also affects the elasticity of the aorta, protruding aortic atheroma, and diastolic coronary flow. Notably, helical blood flow, vortical blood flow, and blood eccentricity assessed by 4D flow CMR became stronger as the disease progressed in patients with AS [13]. Furthermore, a higher grade of helical blood flow would lead to higher EL and WSS distribution in the AAo [30].

In this study, the degree of helical blood flow was higher in patients with AS than in the non-AS control group; however, there was no significant difference in vortical blood flow. Furthermore, we found a significant decrease in helical blood flow, but not in vortical blood flow, after TAVR. Generally, as the diameter of the AAo and age increase, vortical blood flow in the AAo increases [31]. The loss of arterial wall elasticity and the large difference in blood flow velocity between the outside and inside due to dilation of the AAo are thought to cause an increase of vortical blood flow. The AAo diameter of the control subjects was larger than that of the patients with AS but might not produce a significant difference in vortical blood flow. This suggests that vortical blood flow may be greatly affected by the properties of the AAo. On the other hand, helical blood flow also developed in the non-dilated AAo [18] and its grade was significantly associated with the severity of AS resulting from aortic valve calcific fusion and reduced mobility [32]. Therefore, helical rotation in blood flow rather than vortical flow might be reduced after TAVR.

In this study, the eccentricity observed before TAVR remained considerable after TAVR. Florian et al. reported that blood flow eccentricity on 4D flow CMR remains strong even after stented bioprosthesis SAVR [14]. This finding may reflect the blood flow dynamic consequences caused by the rigid opening mechanism and the asymmetric orifices. This may be explained by differences in the opening mechanism and in the sensibility to co-factors such as the angle between the open cusp and the annular plane [33]. Furthermore, Trauzeddel et al. also reported that patients who undergo TAVR show marked eccentricity compared to healthy controls [15]. The characteristics of the implanted stented bioprosthesis may explain the presence of eccentricity after TAVR. Furthermore, it is known that the angle and height of placement for the annular plane differs depending on the type of TAVR. Although eccentricity may vary, we did not have sufficient sample size for a subgroup analysis to determine the differences in blood flow dynamics between balloon- and self-expanding bioprostheses.

### Wall shear stress

Disorganized blood flow patterns such as helical and vortical flow due to abnormal valve opening resulted in markedly altered regional and global WSS in the AAo [34]. Furthermore, elevated WSS in the AAo has been attributed to changes in endothelial function and vascular remodeling in patients with a bicuspid aortic valve and degenerative AS [34, 35]. Our patients showed regional and global decreases in WSS after TAVR, but it did not reach the level of non-AS control subjects. TAVR reduced WSS in the right posterior wall of the middle AAo and the anterior wall of the distal AAo, which are strongly aggravated by increased counterclockwise helical blood flow that passes through the stenotic aortic valve. This finding is consistent with the findings of von Knobelsdorff-Brenkenhoff et al. [13]. They reported a decrease in helical blood flow and eccentricity after TAVR, which altered WSS localization and reduced global WSS in the AAo, suggesting that TAVR decreases WSS and reduces the risk of future aortic disease such as aortic dissection and aneurysm. In contrast, Farag et al. demonstrated that patients who underwent TAVR showed an increase in WSS in the AAo when compared to age- and gender- matched controls [16]. Importantly, WSS decreased after TAVR but did not reach a normal level in our study population. The mechanisms for higher WSS in TAVR patients compared to control subjects could be explained as follows. First, TAVR prosthetic valves are implanted inside a calcified, native aortic valve annulus. This causes the TAVR valve to have a smaller EOAI than that of control subjects. Indeed, in our study, EOAI after TAVR was 1.1 ± 0.2 cm^2^/m^2^, which was smaller than the normal EOAI of 1.8 cm^2^/m^2^. This may provide an explanation as to why WSS post-TAVR did not recover to control level. Second, LV hypertrophy progresses to overcome the pressure load of the stenotic aortic valve. Therefore, stroke volume is higher in patients who undergo TAVR than in healthy subjects [16].

### Energy loss

Our study showed that patients with AS had significantly higher systolic EL in the AAo than non-AS controls. Furthermore, systolic EL in the AAo was significantly reduced after TAVR. Using 4D flow CMR to conduct non-invasive estimations of EL might be useful for assessing hemodynamics in patients with AS, because it more closely reflects the amount of LV energy that is lost during systole due to the obstruction derived from the calcified aortic valve. EL in the AAo is a key indicator of LV afterload, which can worsen LV function [18]. In fact, it is higher in patients with AS than in healthy subjects, suggesting that high EL reflects an increased LV afterload due to abnormal blood flow patterns in the AAo [18, 32]. These findings indicate that TAVR provides efficient blood flow dynamics and can reduce LV afterload.

Interestingly, we also found that EL had a significant negative correlation with EOAI after TAVR. However, there was no significant correlation between EL and AVAI before TAVR in patients with AS. One possible explanation is that most patients had an AVAI < 0.6 cm^2^/m^2^ before TAVR, and the range of AVAI values was small. The patient prosthesis mismatch is described as the mismatch between the hemodynamics derived from a prosthetic valve and the patient’s requirements for adequate cardiac output. Patient prosthesis mismatch is defined by the EOAI, and approximately 37% of patients after TAVR have moderate or severe patient prosthesis mismatch [36]. Severe patient prosthesis mismatch after TAVR leads to subsequent adverse events such as worsening of HF or death [36]. These adverse events may be associated with increased EL, which is a result of a small EOAI and consistent abnormal blood flow patterns after TAVR. In patients with severe AS who undergo TAVR, 4D flow CMR helps to visualize and assess the changes of blood flow dynamics.

### Clinical implications

Abnormal blood flow pattern in the AAo increases LV afterload as energy is dissipated by frictional losses associated with various blood flow phenomena and the aortic wall [18, 32]. As a consequence, accurate assessment of blood flow patterns in patients undergoing TAVR may allow estimation of the risk of TAVR-related adverse events represented by worsening HF due to increased LV afterload. It is noteworthy that TAVR prosthetic valves have been reported to obtain a larger EOAI than those in SAVR. Furthermore, self-expanding valves could achieve a larger EOAI than balloon-expanding valves, and it is necessary to carefully consider which valve to use for patients with a narrow annulus [3, 37]. Obtaining a larger EOAI may lead to an improved prognosis by avoiding the increase in EL. Moreover, Bahlmann et al. reported that EL was superior to EOA in predicting adverse clinical outcomes in patients with moderate to severe AS [38]. Therefore, further studies investigating whether impaired blood flow dynamics including flow patterns, WSS and EL assessed by 4D flow CMR are associated with adverse events after TAVR, or these assessments are useful for selecting the type of prosthetic valve in AS patients undergoing TAVR, are warranted.

### Study limitations

There are several potential limitations that should be acknowledged. First, the spatial and temporal resolution of 4D flow CMR in this study was relatively low. In the setting of spatial and temporal resolution, there is a trade-off between scan time and the accuracy of parameters such as the flow rate, WSS, and oscillatory shear index estimation [39]. Decreasing the spatial and temporal resolution in order to shorten the scan time affects the accuracy of flow quantification and visualization adversely [40] and leads to underestimation of WSS [11, 39, 40]. As the voxel size increases, the accuracy of WSS estimates decreases [41]. Since we favored the accuracy of WSS, we used an optimized, small-size in-plane spatial resolution (1.6 × 1.8 mm^2^). To minimize the influence of anisotropic voxels, we set the slice gap to 2–2.5 mm. In our preliminary study with young healthy volunteers (n = 5), the scan time was much longer for 20 phases (12.3 ± 1.9 min) than for 12 phases (6.6 ± 0.6 min). In elderly patients with severe heart disease, longer scan duration was impractical, and we decided to reduce the temporal resolution. The lower temporal resolution might imply the loss of possible information contained in other systolic phases [42]. However, in this study, all acquisitions were made with the same imaging parameters and analyzed with the same methodology both pre- and post-TAVR. Since our study focused on changes in parameters between pre- and post-TAVR, underestimation of WSS might be compensated. Compressed sensing (CS), which exploits the inherent compressibility of CMR data, has been combined with parallel imaging to achieve even higher acceleration rates, enabling 4D flow CMR scan times to become clinically feasible [43, 44]. Ma et al. applied CS to 4D flow imaging of the thoracic aorta and achieved a scan time of 2 min [45]. However, CS 4D flow CMR has been reported to underestimate peak flow and velocity. Further investigation using CS-based protocols is needed. Second, since our CMR unit did not allow multiple VENC settings, we set a single VENC value according to the velocity in the head/foot direction. However, this setting could measure the majority of high velocity compared to anterior/posterior or right/left directions. Third, the Reynolds number could provide more information on the impact of turbulent flow in the aorta from the stenotic aortic valve and how it affects the calculation of the parameters using 4D flow CMR. However, we were unable to actually calculate the Reynolds number due to the inability to measure aortic inlet diameter by 4D flow CMR and to accurately evaluate flow velocity after TAVR due to signal loss caused by metal artifacts of the implanted valve. Therefore, we calculated the substitute Reynolds number for each case using the diameter of the AAo on computed tomography, mean aortic velocity on echocardiography, and blood kinematic viscosity ν = 3.3 × 10^−6^. Substitute Reynolds number decreased significantly from 9741 [8992–12,574) before TAVR to 4358 [3688–4790] after TAVR (P < 0.001). From these numbers, because the WSS calculation was done in a turbulent state in both pre- and post-TAVR, we think that the comparison of WSS is valid. Furthermore, because the WSS is calculated by directly deriving the blood flow velocity gradient from the flow velocity near the wall in the software used for this study, the calculation is applicable in both laminar and turbulent flows. Generally, in 4D flow CMR, turbulent flow decreases the signal and causes an underestimation of the flow velocity [46]. Due to the limitations of the spatial resolution of 4D flow CMR, the reliability of the absolute value of WSS is controversial, however, we believe that it is meaningful in comparing relative changes before and after the intervention. We recognize that the failure to assess the actual effect of the Reynolds number on the changes of blood flow dynamics in 4D flow CMR is a major limitation of the present study. Fourth, although the blood flow patterns were evaluated after thorough confirmation of the evaluation methods by two radiologists and one cardiologist, these evaluations may have differed among them. This is considered to be a limitation of the visual semi-quantitative method. In this study, those assessments were reproducible (but not yet well standardized) and susceptible to observer bias. Fifth, we did not have sufficient sample size for a subgroup analysis to determine the differences in blood flow dynamics between balloon- and self-expanding bioprostheses. Although our findings showed that the self-expanding bioprostheses might reduce the WSS and EL in the AAo more than the balloon-expanding bioprostheses, further studies are warranted to confirm these findings. Finally, the number of adverse events and the sample size were too small to evaluate changes in the LV function or remodeling. Therefore, we were unable to confirm the relationship between impaired blood flow dynamics (obtained from 4D flow CMR) and worse clinical outcomes. Accordingly, further larger-scale studies are necessary to confirm the prognostic significance of the changes in blood flow dynamics in patients who undergo TAVR.

## Conclusions

In severe AS patients who undergo TAVR, 4D flow CMR is useful for visualizing and assessing the changes of blood flow dynamics. TAVR improves blood flow dynamics especially when a larger EOAI is obtained.

## Supplementary Information

**Additional file 1: Video S1.** In a patient, marked vortical flow was observed before TAVR (MP4 675 KB)

**Additional file 2: Video S2.** Vortical flow decreased after TAVR. In another patient

**Additional file 3: Video S3.** Marked helical flow was observed before TAVR

**Additional file 4: Video S4.** Helical flow disappeared after TAVR. TAVR indicates transcatheter aortic valve replacement

**Additional file 5.** 

## Data Availability

The datasets used during the current study are available from the corresponding author on reasonable request.

## Abbreviations

3D: Three-dimensional
4D: Four-dimensional
AAo: Ascending aorta
AS: Aortic stenosis
AVA: Aortic valve area
AVAI: Aortic valve area index
CMR: Cardiovascular magnetic resonance
CS: Compressed sensing
EL: Energy loss
EOA: Effective orifice area
EOAI: Effective orifice area index
HF: Heart failure
LA: Left atrium/left atrial
LAVI: Left atrial volume index
LV: Left ventricle/left ventricular
LVEDD: Left ventricular end-diastolic dimension
LVEF: Left ventricular ejection fraction
LVESD: Left ventricular end-systolic dimension
LVM: Left ventricular mass
LVMI: Left ventricular mass index
PG: Pressure gradient
RWT: Relative wall thickness
SAVR: Surgical aortic valve replacement
TAVR: Transcatheter aortic valve replacement
VENC: Velocity encoding
V_max_: Maximal velocity
WSS: Wall shear stress
