# Supplementary material for: Blood flow dynamics with four-dimensional flow cardiovascular magnetic resonance in patients with aortic stenosis before and after transcatheter aortic valve replacement
Source: J Cardiovasc Magn Reson. 2021 Jun 28;23:81. doi: 10.1186/s12968-021-00771-y (PMC8237445; doi:10.1186/s12968-021-00771-y)
Supplement: Supplementary file 5 — Additional file 5. [file 12968_2021_771_MOESM5_ESM.docx]

| **Supplementary Table 1** Parameters on 4D flow CMR at post- TAVR between very severe and severe AS | | | |
| --- | --- | --- | --- |
| Variables | **Very severe AS**  n = 18 | **Severe AS**  n = 14 | ***P* value** |
| Vortical flow | 1.8 ± 0.8 | 1.9 ± 0.7 | 0.70 |
| Helical flow | 1.5 ± 0.6 | 1.3 ± 0.5 | 0.32 |
| Flow eccentricity | 2.6 ± 0.6 | 2.8 ± 0.4 | 0.42 |
| WSS average, Pa | 6.0 (5.6 – 6.7) | 6.1 (5.0 – 7.1) | 0.88 |
| WSS peak, Pa | 51.2 (38.8 – 53.7) | 46.7 (38.0 – 49.8) | 0.40 |
| EL LV diastole, mW | 5.1 (3.7 – 8.9) | 8.4 (7.1 – 17.8) | 0.139 |
| EL LV systole, mW | 4.8 (2.6 – 7.1) | 8.1 (2.8 – 9.6) | 0.34 |
| EL AAo diastole, mW | 7.4 (3.6 – 14.8) | 9.0 (5.6 – 12.5) | 0.72 |
| EL AAo systole, mW | 13.5 (10.8 – 30.3) | 17.5 (11.8 – 22.9) | 0.82 |

Continuous variables are presented as mean ± standard deviation if normally distributed, and median (interquartile range) if not normally distributed

4D four-dimensional, AAo, ascending aorta; AS, aortic stenosis; CMR, cardiovascular magnetic resonance; EL, energy loss; LV, left ventricle; TAVR, transcatheter aortic valve replacement; WSS, wall shear stress

| **Supplementary Table 2** Baseline characteristics of patients with AS and control | | | |
| --- | --- | --- | --- |
| Variables | **Study population**  n = 32 | **Non-AS Control**  n = 12 | ***P* value** |
| Age, years | 82 ± 5 | 82 ± 6 | 0.83 |
| Male, n (%) | 17 (53) | 9 (75) | 0.189 |
| Body mass index, kg/m^2^ | 21.3 ± 3.8 | 22.6 ± 4.1 | 0.38 |
| LVEDD, mm | 46.7 (43.0 – 50.0) | 48.5 (41.3 – 52.0) | 0.66 |
| LVESD, mm | 28.0 (24.5 – 34.5) | 33.5 (27.6 – 42.0) | 0.129 |
| LVEF, % | 61 (54 – 70) | 59 (48 – 64) | 0.190 |
| E/A ratio | 0.70 (0.56 – 0.97) | 0.82 (0.70 – 1.03) | 0.087 |
| E/e’ ratio | 13.1 ± 4.8 | 12.7 ± 4.8 | 0.73 |
| LAVI, mL/m^2^ | 45.5 (39.7 – 63.0) | 44.9 (34.6 – 51.7) | 0.41 |
| LVMI, g/m^2^ | 117.5 (102.0 – 141.0) | 105.2 (90.4 – 112.3) | 0.031 |
| Ascending aortic diameter, mm | 33.2 ± 3.7 | 35.1 ± 2.1 | 0.058 |

Continuous variables are presented as mean ± standard deviation if normally distributed, and median (interquartile range) if not normally distributed

Categorical variables are presented as number of patients (%)

AS, aortic stenosis; LAVI, left atrial volume index; LVEDD, left ventricular end-diastolic dimension; LVESD, left ventricular end-systolic dimension; LVEF, left ventricular ejection fraction; LVMI, left ventricular mass index

| **Supplementary Table 3** Parameters on 4D flow CMR between the study population at pre-/post-TAVR and the control group | | | | | |
| --- | --- | --- | --- | --- | --- |
| Variables | **Control**  n = 12 | **Study population at pre-TAVR**  n = 32 | ***P* value**  vs. control | **Study population at post-TAVR**  n = 32 | ***P* value**  vs. control |
| Vortical flow | 1.7 ± 0.7 | 1.7 ± 0.8 | 0.89 | 1.9 ± 0.8 | 0.42 |
| Helical flow | 1.1 ± 0.3 | 1.9 ± 0.8 | 0.002 | 1.4 ± 0.6 | 0.061 |
| Flow eccentricity | 2.0 ± 1.0 | 2.6 ± 0.5 | 0.050 | 2.7 ± 0.5 | 0.017 |
| WSS average, Pa | 4.2 (3.2 – 5.3) | 6.7 (6.1 – 8.4) | < 0.001 | 6.0 (5.4 – 7.0) | 0.002 |
| WSS peak, Pa | 35.9 (27.8 – 42.9) | 52.0 (45.6 – 62.2) | < 0.001 | 47.5 (38.4 – 53.1) | 0.003 |
| EL LV diastole, mW | 4.0 (2.1 – 11.6) | 5.4 (3.2 – 10.5) | 0.34 | 6.7 (3.7 – 14.9) | 0.21 |
| EL LV systole, mW | 3.6 (2.3 – 7.9) | 4.4 (2.6 – 7.9) | 0.72 | 5.2 (2.6 – 9.6) | 0.48 |
| EL AAo diastole, mW | 4.1 (2.2 – 8.3) | 7.4 (5.1 – 10.5) | 0.078 | 8.0 (4.9 – 14.8) | 0.062 |
| EL AAo systole, mW | 6.4 (5.5 – 18.8) | 25.8 (18.6 – 36.2) | < 0.001 | 15.6 (10.8 – 25.1) | 0.066 |

Continuous variables are presented as mean ± standard deviation if normally distributed, and median (interquartile range) if not normally distributed

4D, four-dimensional; AAo, ascending aorta; CMR, cardiovascular magnetic resonance; EL, energy loss; LV, left ventricle; TAVR, transcatheter aortic valve replacement; WSS, wall shear stress

| **Supplementary Table 4** Linear regression analysis of AVAI or EOAI and 4D flow CMR parameters at pre-/post-TAVR | | | | |
| --- | --- | --- | --- | --- |
|  | Variables | **R^2^** | **Coefficient (95% CI)** | ***P* value** |
| Pre-TAVR |  |  |  |  |
| AVAI | WSS average | 0.074 | 5.21 (-0.50 – 10.92) | 0.072 |
|  | WSS peak | -0.032 | -9.08 (-88.69 – 70.54) | 0.82 |
|  | EL AAo diastole | -0.034 | 0.95 (-29.19 – 31.08) | 0.95 |
|  | EL AAo systole | 0.038 | 69.11 (-26.79 – 165.02) | 0.151 |
| Post-TAVR |  |  |  |  |
| EOAI | WSS average | 0.084 | -2.06 (-4.20 – 0.09) | 0.059 |
|  | WSS peak | 0.067 | -14.07 (-30.10 – 1.95) | 0.083 |
|  | EL AAo diastole | 0.041 | -11.13 (-26.19 – 3.94) | 0.142 |
|  | EL AAo systole | 0.116 | -30.52 (-58.59 – -2.45) | 0.034 |

4D, four-dimensional; AAo ascending aorta; AVAI, aortic valve area index; CI, confidence interval; CMR, cardiovascular magnetic resonance; EL, energy loss; EOAI, effective orifice area index; TAVR, transcatheter aortic valve replacement; WSS, wall shear stress

| **Supplementary Table 5** Parameters on 4D flow CMR at post-TAVR and EOAI between the self- and the balloon-expanding valves | | | |
| --- | --- | --- | --- |
| Variables | **Self-expanding**  n = 6 | **Balloon-expanding**  n = 26 | ***P* value** |
| Vortical flow | 1.7 ± 0.5 | 1.9 ± 0.8 | 0.50 |
| Helical flow | 1.5 ± 0.5 | 1.4 ± 0.6 | 0.55 |
| Flow eccentricity | 2.7 ± 0.5 | 2.7 ± 0.5 | 0.81 |
| WSS average, Pa | 5.8 (3.9 – 7.4) | 6.1 (5.6 – 7.0) | 0.148 |
| WSS peak, Pa | 50.3 (35.4 – 51.5) | 46.9 (38.8 –53.4) | 0.74 |
| EL LV diastole, mW | 7.6 (0.9 – 14.9) | 6.6 (3.7 – 14.7) | 0.79 |
| EL LV systole, mW | 5.8 (1.2 – 11.2) | 5.1 (2.6 – 8.8) | 0.91 |
| EL AAo diastole, mW | 5.8 (1.1 – 14.8) | 8.0 (5.0 – 12.5) | 0.40 |
| EL AAo systole, mW | 11.5 (2.3 – 20.3) | 15.6 (11.8 –25.1) | 0.21 |
| EOAI, cm^2^/m^2^ | 1.12 ± 0.19 | 1.12 ± 0.24 | 0.88 |

Continuous variables are presented as mean ± standard deviation if normally distributed, and median (interquartile range) if not normally distributed

4D, four-dimensional; AAo, ascending aorta; CMR, cardiovascular magnetic resonance; EL, energy loss; EOAI, effective orifice area index; LV, left ventricle; TAVR, transcatheter aortic valve replacement; WSS, wall shear stress

| **Supplementary Table 6** The amount and rate of changes on 4D flow CMR parameters before and after TAVR between the self- and the balloon-expanding valves | | | |
| --- | --- | --- | --- |
| Variables | **Self-expanding**  n = 6 | **Balloon-expanding**  n = 26 | ***P* value** |
| **Amount of changes** |  |  |  |
| WSS average, Pa | -3.1 (-3.8 – -2.3) | -0.3 (-2.1 – 0.5) | 0.011 |
| WSS peak, Pa | -17.5 (-35.6 – -1.1) | -2.9 (-16.4 – 5.2) | 0.122 |
| EL LV diastole, mW | 0.1 (-5.8 – 4.8) | 1.2 (-1.6 – 5.2) | 0.67 |
| EL LV systole, mW | -0.4 (-4.8 – 2.6) | 0.1 (-1.5 – 3.2) | 0.59 |
| EL AAo diastole, mW | -4.2 (-9.0 – 0.2) | -0.2 (-2.1 – 4.3) | 0.110 |
| EL AAo systole, mW | -23.6 (-37.6 – -10.6) | -10.0 (-18.1 – -2.1) | 0.089 |
| **Rate of changes** |  |  |  |
| WSS average | -0.35 (-0.38 – -0.34) | -0.04 (-0.28 – 0.08) | 0.008 |
| WSS peak | -0.25 (-0.41 – -0.03) | -0.06 (-0.25 – 0.11) | 0.162 |
| EL LV diastole | 0.14 (-0.69 – 1.08) | 0.30 (-0.24 – 0.70) | 0.59 |
| EL LV systole | -0.23 (-0.66 – 0.63) | 0.05 (-0.30 – 0.97) | 0.31 |
| EL AAo diastole | - 0.38(-0.85 – -0.12) | -0.03 (-0.26 – 0.93) | 0.110 |
| EL AAo systole | -0.67 (-0.91 – -0.37) | -0.43 (-0.58 – -0.08) | 0.064 |

Continuous variables are presented as median (interquartile range)

4D, four-dimensional; AAo, ascending aorta; CMR, cardiovascular magnetic resonance; EL, energy loss; LV, left ventricle; TAVR, transcatheter aortic valve replacement; WSS, wall shear stress
